# Perspectives on Frailty Among Emergency Physicians: A Qualitative Descriptive Study

**DOI:** 10.7759/cureus.75670

**Published:** 2024-12-13

**Authors:** James Smyth, Joanne Dollard, Renuka Visvanathan, Mandy Archibald

**Affiliations:** 1 Adelaide Medical School, The University of Adelaide, Adelaide, AUS; 2 College of Nursing, University of Manitoba, Winnipeg, CAN

**Keywords:** emergency medicine, emergency services, frail older adult, frailty, qualitative research

## Abstract

Background

The proportion of older people in the general population is rising. Accompanying this rise is an increased prevalence of frailty. Frailty is a syndrome of increased vulnerability to stressors due to decreased physiological reserve and is linked to increased health services use. Frailty assessment provides a comprehensive insight into an older person’s health status but is not regularly performed in emergency departments (EDs) worldwide. Understanding emergency physicians’ (EPs) perspectives on frailty could assist in facilitating the implementation of frailty assessments in the ED. However, little is known about EP perceptions of frailty.

Objectives

The aim of this study was to explore perspectives on frailty among EPs.

Design

An exploratory qualitative descriptive study was conducted. EPs’ perspectives on frailty were explored in semi-structured interviews, including questions on the meaning of frailty, its detection, management, and potential to improve the care of frail ED patients. Interviews were recorded and transcribed.

Setting

Central Adelaide Local Health Network, which has two EDs in Adelaide, South Australia.

Participants

Sixteen EPs were interviewed. Eleven (69%) were male EPs and 10 (63%) were practicing as EPs for 4 years or less.

Measurements

We analyzed interview transcripts by inductive thematic analysis. We generated, iteratively revised, and applied a coding framework, enabling progression to the formulation of themes.

Results

We developed three themes. Theme 1: EPs did not perceive frailty as a priority of care due to their focus on acuity, and lack of knowledge, time, and resources. Theme 2: EPs referred to detecting and managing frailty indirectly rather than formally. Theme 3: EPs saw a beneficial role in frailty recognition and management in the future.

Conclusions

EPs did not perceive frailty as a priority of care. However, they had a positive view on progressing with increased frailty awareness and holistic management in EM, for patient and health system benefits.

## Introduction

Frailty is a syndrome of increased vulnerability to acute stressors, such as illness, resulting in a heightened risk of adverse outcomes such as falls and impaired quality of life [1]. Increased health service utilization, such as emergency department (ED) visits, is a consequence of frailty [2]. The frailty burden is increasing with the growing aging population worldwide. For example, in Australia, the prevalence of frailty among people aged 65 years and over is projected to rise to 609,306 people by 2027 (from 415,769 in 2016); this trend will have significant quality of life, economic and political implications [3].

Contrary to common belief [4,5], frailty status is non-static and may be reversed if detected early [6]. Measures such as improved nutrition, treatment of comorbidities, and exercise to prevent or reverse the progression of frailty can lead to beneficial outcomes, such as improved mobility and decreased risk such as with falls and injury [1]. Frailty assessment is important as it provides insight into an older person’s health status [1,7], and thereby enables its early detection and timely intervention for possible reversal or stabilization.

An ED presentation can be a significant point in a frail older patient’s journey, irrespective of whether they are admitted or discharged [8]. Importantly, interest is increasing in frailty assessment in the ED due to its potential contribution in guiding clinical decision-making, formulating prognoses, and as a trigger for advance care planning [2, 9-11]. However, variable rates of frailty assessment in EDs have been reported, suggesting underlying barriers to its uptake [12-14]. For instance, a 2022 audit survey in the United Kingdom found that frailty assessments in ED were being conducted in 50 (62.5%) of 80 hospitals reporting a policy to perform frailty screening [13]. Possible barriers to attention to frailty in acute care include lack of knowledge, focus on emergency issues, and lack of resources [15-17].

In a recent scoping review, we reported gaps in evidence regarding the relationship between emergency physician (EP) perspectives (i.e. ED senior doctors or consultants) on frailty and the perceived role of frailty assessments in EDs [18]. In addition, there is little known about EPs’ perspectives on frailty itself. There have been at least two interview studies on frailty involving EPs within groups of healthcare workers (HCWs) [15,16]; however, the perspectives attributable to EPs subgroups cannot be fully determined within these mixed samples. Our research aimed to address this gap by focusing exclusively on EPs’ perspectives on frailty given their role as leaders at the ‘front door’ of hospitals and the consequent need to consider their perspectives when developing frailty interventions for the ED.

This study is part of a protocol-guided research program set in 2017 in the context of a relative dearth then of knowledge relating to the perspectives of different HCW groups (including general practitioners (GPs), practice nurses, and orthopedic surgeons) as well as consumers, on frailty and frailty screening [19]. The insight into these stakeholder groups’ perspectives was deemed an important step in improving awareness and implementing interventions to identify and manage frailty; related findings have been published [5,17,20]. The aim of this study was to understand the perspectives of EPs on frailty as a unique contribution to this program of work. The findings of this article were presented at the Australasian College for Emergency Medicine (ACEM) Annual Scientific Meeting in Canberra on November 23, 2023. The perspectives of EPs on frailty screening in the ED are the focus of a future paper as part of this program of research.

## Materials and methods

We used an exploratory qualitative descriptive method, which represents a straight approach to sampling, data collection, and analysis [21-24]. Qualitative description includes a low inference approach to the analysis of participants’ interview data, with the identification of patterns and concepts that stays close to participants’ words and perspectives. This leads to an inductive development of codes and themes [21-24]. The process was appropriate and comprehensive because relatively little was known about the topic. We followed the method in our published research protocol [19]. The consolidated criteria for reporting qualitative research (COREQ) guided our reporting [25].

Setting

The study was conducted in the Central Adelaide Local Health Network (CALHN) which encompasses two major EDs, at the Royal Adelaide Hospital and The Queen Elizabeth Hospital (TQEH). CALHN serves a catchment area of 464,692 people [26].

Participants and recruitment

The ED directors agreed for JS (lead author) to invite EPs to participate in the study. There were approximately 50 EPs working in the CALHN EDs. The study was promoted via introductory briefings by email, paper-based circulation of a study information flyer, and in-person discussions. The inclusion criterion was being an EP (consultant in emergency medicine (EM)) working in the CALHN EDs. The interviewer (JS) approached interested EPs and recruited those willing to take part (purposive sampling by meeting the inclusion criterion). A sample of 8 to 16 semi-structured interviews was planned [19], with iterative data analysis occurring. We aimed to reach data saturation in the course of the interview program, i.e., when no new conceptual codes were identified.

Interviews and data collection

Single interviews with participants (while not on clinical duty) were scheduled at mutually convenient times, between May 2018 and May 2019. JS conducted all 16 interviews, face to face, at TQEH ED in a relatively quiet and comfortable room with minimal disruption. The setting away from the emergency clinical floor enabled the interviews to proceed without interruption and distraction. After receiving an introductory briefing and giving written informed consent, participants provided demographic information (year of birth, gender, number of years in practice as an EP). Interview questions followed a semi-structured guide, were open-ended, and addressed EP perspectives on features of frailty, its detection and management as well as frailty screening in EDs (Appendix). Interviews were audio-recorded. JS wrote field notes about impressions formed in the interview. JS transcribed (verbatim) the 16 audio recordings in Microsoft Word documents.

Reflexivity

JS was a colleague of the participants, working as an EP in CALHN. Therefore, JS was reflexive in the active process of developing self-awareness of his knowledge and expertise on frailty, as well as how his insider position could influence recruitment, data collection, transcription, analysis, and interpretation [27]. JS wrote reflexive notes throughout the study, asked critical questions to challenge his assumptions, and discussed reflexivity within research meetings. Reflexivity also assisted with the analysis, and collaboration with co-researchers by maintaining an open-minded perspective in the development of themes and relating to participants.

Data analysis

An inductive and iterative thematic analysis of the interview data corpus using qualitative description was undertaken [19, 21]. Memos and annotations were written from an early stage of analysis to substantiate the development of concepts, codes, and themes, as well as maintain an audit trail [19, 28, 29]. The data analysis was managed in NVIVO software (version 12.7.0). Participant demographic data were analyzed in Excel (version 16.66.1). Two authors (JS and JD) independently analyzed six transcripts to develop, review, and discuss coding; JS analyzed the remaining ten transcripts. A coding framework was developed through codebooks including the definition of codes, and iteratively refined as coding progressed, leading to the formulation of themes and their sub-themes. The investigators held serial meetings on the coding and theme developments, with JS rereading transcripts and relistening to audio files to review and update interpretation when indicated. Themes were linked to codes formulated by qualitative description with attention to the frequency and significance of supporting data relating to the research question on the perspectives of EPs on frailty. JS drafted diagrams as mind maps to help develop the themes and sub-themes. Quotations provided underlying evidence for themes.

Ethics approval

The University of Adelaide Human Research Ethics Committee granted approval (HR-2016-238) to conduct the study.

## Results

All 16 EPs approached consented to participate. Eleven (69%) participants were male. Ten (63%) participants were aged 40 years and older. The median duration of practice as an EP for the group was 3 years within a range of 4 months to 15 years; 10 (63%) were practicing as EPs for 4 years or less. The 16 interviews ranged from 10 to 41 minutes in duration (mean = 20 minutes). After analysis of the 12th interview, we observed no new codes being developed from the final four interviews, confirming an impression of data saturation. Three themes with related sub-themes were developed (Table 1).

**Table 1:** Themes and Sub-themes ED: emergency department; EM: emergency medicine; EPs: emergency physicians

Themes	Sub-themes
Theme 1: EPs did not perceive frailty as a priority of care due to their focus on acuity, and lack of knowledge, time, and resources.	Sub-theme 1.1: EPs viewed patients’ acute problems as the immediate priority.
	Sub-theme 1.2: EPs perceived a gap in their knowledge about frailty and infrequently used the term.
	Sub-theme 1.3: EPs identified time and workload pressures in the ED contributed to prioritizing acute problems.
	Sub-theme 1.4: EPs perceived a lack of resources to address frailty.
Theme 2: EPs referred to identifying and managing frailty indirectly rather than formally.	Sub-theme 2.1: EPs understood frailty as a state of functional decline, usually in older age with multiple factors and vulnerability.
	Sub-theme 2.2: EPs were guided by clinical skills and gestalt to recognize frailty.
	Sub-theme 2.3: EPs perceived multidisciplinary care as key to addressing frailty-related problems, though not necessarily by the ED team.
Theme 3: EPs saw a beneficial role in ED frailty recognition and management in the future.	Sub-theme 3.1: EPs perceived potential benefits of frailty awareness in EM.
	Sub-theme 3.2: EPs advocated for resources and training related to frailty.

Theme 1: EPs did not perceive frailty as a priority of care due to their focus on acuity, and lack of knowledge, time, and resources.

EPs did not see frailty as a priority for care in the ED, perceiving patients’ acute problems as the priority before chronic problems. They also acknowledged a relative lack of formal or explicit knowledge of frailty. Participants perceived time and workload pressures as well as lack of resources as barriers to addressing frailty in the ED.

Sub-theme 1.1: EPs Viewed Patients’ Acute Problems as the Immediate Priority.

Most of the participants perceived their focus and orientation were primarily on patients’ acute problems. EPs considered that chronic problems such as frailty took time and were a diversion from acute care. As one participant stated: ‘*You’re often trying to take care of the acute problem, even in the frail... and… to take the time away … and work these more chronic issues is a bit harder*’ (EP 13). A minority referred to attending to holistic or comprehensive care of frail patients in the ED. As one interviewee said: ‘*…a holistic assessment of how their frailty is affecting them and their functioning in their life…*’ (EP 11) and discussed how this should be prioritized when considering frailty assessment.

*Sub-theme 1.2: EPs Perceived a Gap in Their Knowledge about Frailty and Infrequently Used the Term*.

Most participants perceived gaps in their knowledge about frailty. Some referred to frailty and other problems in geriatric medicine not being addressed thoroughly in their training. This contributed to their view that frailty was not a priority in ED patient care. One participant commented on the status in EM practice about problems associated with aging not having been anticipated: ‘*It’s probably not something I know a whole lot about;...it’s something that’s not focussed on a great deal in our training … I don’t think we envisaged the situation that we’re in now with an ageing population and all the problems related to ageing*’ (EP 9).

Most participants stated they seldom used the term frailty in EM practice in oral and written communication. A minority referred to thinking about the concept in contrast to explicitly speaking or writing about it. Some also expressed a commitment to considering the clinical elements of frailty as opposed to the conceptual syndrome: ‘*You need an actual observation of …the changes*’ (EP 4). ‘*it’s not a concept that I would routinely use in my clinical practice, perhaps because our decision making within ED …, often has to be more concrete than referring to... the concept of frailty…*’ (EP 16). One participant described using the term as part of the assessment of older patients when considering risks with ED discharge: ‘*where I’ve got some concern about going home, about their safety on discharge*’ (EP 11).

Sub-theme 1.3: EPs Identified Time and Workload Pressures in the ED Contributed to Prioritizing Acute Problems.

Participants perceived operational factors such as high patient activity and workloads (e.g., with ED overcrowding), contributed to experiencing time pressures. They felt the latter contributed to prioritizing acute problems over frailty. As one respondent said: ‘*just because of the time factor, and the pressure and the number of patients, and the fact we don’t have all day to kind of sort out something that is probably… not acutely the issue today*’ (EP 10). Similarly, participants also referred to time constraints, associated with limited patient contact and length of stay in the ED, reinforcing EP’s focus on addressing acute over chronic problems. As one participant expressed: ‘*our focus is nowadays more on time, just get them out … from the emergency quickly treated if there is any acute intervention but frailty is … a chronic condition …*’ (EP 12).

*Sub-theme 1.4: EPs Perceived a Lack of Resources to Address Frailty*.

Participants identified resource issues as barriers to addressing frailty as a priority in the ED. The most common issue noted was the difficulties in accessing ancillary staff to provide support for patients with frailty in the ED and the community. As one expressed: ‘*and providing community services per se is not a focus that EPs have but certainly we do advocate for but haven’t the actual resources available*’ (EP 7). Single respondents referred to, as obstacles in the care of frail older persons, ED staff shortages (EP 9): ‘*There is a lot of overcrowding and things you know are quite crazy … in terms of workload, staff ratios and things like that*’, and problems with the physical environment such as small signs and not transferring older patients to the appropriate ward (EP 3): ‘*Most of the EDs I’ve worked in aren’t really geared up for elderly patients such as simple things like signage being small…They may not be admitted to the right ward …*’.

Theme 2: EPs referred to identifying and managing frailty indirectly rather than formally.

Participants understood frailty as a functional decline mainly in older age combined with multiple factors and risks. They perceived the progress of frailty could be delayed, and less often reversed. Participants described detecting frailty indirectly using clinical observations and gestalt, as opposed to formal screening and assessment. Participants recognized the importance of multidisciplinary care and communication. Without formal identification of frailty, they referred to the management of patients’ related problems and clinical course by addressing associated factors and risks.

Sub-theme 2.1: EPs Understood Frailty as a State of Functional Decline, Usually in Older Age with Multiple Factors and Vulnerability.

Most EPs had a clinical understanding of frailty as a state of functional decline, mainly associated with aging and multiple other factors, as well as risk and vulnerability. As one participant expressed: ‘*a general …functional decline and impaired ability to function normally in a normal environment*’ (EP 11). A minority of the participants referred directly to a decreased physiological reserve; as one EP stated: ‘*decrease in the physiological state*’ (EP 5). EPs understood there were contributory and associated factors, often in varying combinations. The most common factors contributing to frailty identified, in addition to aging, were physical and mental comorbidities including decreased cognitive ability, physical function, and ability for activities of daily living. As one interviewee described: ‘*those who have an underlying decrease of physical or mental fitness essentially compared to what they were at a younger age or healthier time*’ (EP 4). Less frequently mentioned factors contributing to frailty were falls, social issues, malnutrition, and medications.

Most participants referred to the vulnerability or risk for frail patients to experience an adverse event such as falls, injury, disease, or deterioration. As one respondent expressed: ‘*they are at higher risk for injuries, diseases …*’ (EP 1). The vicious cycle model of frailty, where different factors are linked to the risk of further deterioration, was described by a couple of participants, as illustrated by this quotation: ‘*it can be a vicious cycle when they’re not caring for themselves as well, their diet suffers, and then they leave themselves vulnerable to another insult*’ (EP 9).

About half of the participants alluded to their observations of frail patients’ difficult experiences. They perceived frail patients in the health system as feeling vulnerable and isolated. One respondent referred to the depressing effect of frailty: ‘*I think they feel …lost in the system. They can have a sense of despair…in that they’re battling not only their own physical issues which mentally drags them down*’ (EP 5). One EP referred to patients feeling the term frailty was disparaging and negative: ‘*it might be taken as sort of derogatory…*’ (EP 8).

Participants understood frailty was non-static, relating to the progression of frailty occurring more frequently than delayed onset, reversal, or prevention. Broadly, two rates of progression were alluded to. On one hand, most participants thought that the progression of frailty was usually slow and gradual, with aging as the most frequently described contributory factor. On the other hand, some participants mentioned that frailty could progress faster, such as when individuals experienced an acute event or change of living conditions, like after major trauma, hospitalization, or admission to a nursing home. As one participant said: ‘*it develops insidiously,... except in some circumstances of major trauma and those sorts of things when you can suddenly have a change in circumstance…'* (EP 7).

Participants alluded to different degrees of treatment effectiveness on the trajectory of frailty. Most EPs deemed progression of frailty could be slowed with a multidisciplinary approach addressing the causes and associated factors as indicated, such as exercise, mental and social stimulation, diet, and medications. Most referred to frailty prevention as delaying deterioration. As one EP stated: ‘*regular exercise as you get older, steady diet would probably slow the ageing process or decrease the chance of frailty coming quicker than later*’ (EP 15). Half of the participants referred to potential frailty reversal. As one respondent alluded: ‘*with the right kind of supports and services in place to boost them, support them, they may well be able to come back*’ (EP 7).

Sub-theme 2.2: EPs were Guided by Clinical Skills and Gestalt to Recognize Frailty.

Participants’ identification of frailty was indirect. None of the participants reported formally screening for frailty using screening tools and following with formal assessment and diagnosis of frailty. They described a path of clinical observation skills and gestalt to detect frailty. As one EP stated: ‘*My feeling is that we have a gestalt, a good picture of it*’ (EP 7). Some participants alluded to this process contributing to clinical decision-making such as for investigations and treatment. As one participant said: ‘*One of the things I ask, if someone’s from a nursing home, is if they’re cognitively intact or not, and if they’re not fully cognitively intact what their...limits are... so I already make as a very crude or blunt assessment and that does as such influence see how, assess and manage patients*’ (EP 6).

About half of the participants referred to the need for timely identification of frailty, to follow on with treatment to prevent frailty progression. As one participant exemplified: ‘*I think the most important thing is the early identification and trying to address it before it deteriorates*’ (EP 8). A quarter of the interviewees referred to current under-recognition or decreased awareness of patients in ED having frailty, as referred to in this quotation: ‘*simply under-recognized in the ED*’ (EP 15).

Sub-theme 2.3: EPs Perceived Multidisciplinary Care as Key to Addressing Frailty-related Problems, though not Necessarily by the ED Team.

While perceiving the acute problem as the priority to address in the ED, most participants supported the role of multidisciplinary management of frailty-related problems after priority acute care, though not necessarily in the ED. Most referred to managing multiple associated factors as well as vulnerability. As one EP said: ‘*The next step … is to optimize their health both physically, mentally and socially to help improve their frailty, to make them less frail if physically possible, to prevent re-presentation to the emergency department again*’ (EP 14).

To enable multidisciplinary care for frail patients, EP saw the key role of ED staff communicating across different settings, such as with HCWs in the community and nursing homes. Some participants referred to the leadership role of the general practitioner (GP) for the collaborative follow-up of frail patients in the community. EPs also highlighted the importance of communicating with patients and informal caregivers. As one respondent stated: ‘*it’s more about linking them in …with other services suggesting their primary care provider, the GP refer them to things like the falls clinic, physiotherapist, potentially dietician….It generally requires multidisciplinary input …so potentially it is just raising awareness with the patient and their family members…*’ (EP 6).

Theme 3: EPs saw a beneficial role for frailty recognition and management in EM in the future.

Participants perceived frailty awareness such as frailty screening, assessment, and treatment could contribute to the future development of emergency care for older patients. They recommended the improved provision and use of frailty-related resources.

*Sub-theme 3.1: EPs Perceived Potential Benefits of Frailty Awareness in EM*.

Participants identified the potential benefits of increased attention by HCWs to frailty in the emergency care of frail older patients. Most recognized the potential value of formal frailty screening within the ED patient assessment to improve their awareness of patients with frailty. As one participant exemplified: ‘*if you identify someone afflicted with frailty, then when the screen is initiated, it could be official*’ (EP 11). Participants expressed their responsibility and commitment to progressing with frailty-related patient care. As one EP said: ‘*We’re in the ED, the coalface having to learn how we manage these patients complex medical and social needs*’ (EP 3).

Most participants recognized that awareness of the patient’s frailty status could be used within ED decision-making processes. Attention to frailty in the ED including flagging, assessment, and management planning could contribute to improved comprehensive or holistic care either in the hospital or the community. As one EP said: ‘* … more attention … physical and communicative and cognitive processes*’ (EP 2). This progress with patient-centered care could consequently improve patient outcomes, such as function and quality of life. As one EP stated: ‘*having them supported so that they continue to function and avoid sort of the loss of function and independence and care*’ (EP 16).

Some participants referred to considering the frailty status in evaluations and communication prior to deciding on comfort or palliative care for a patient (when indicated and appropriate). As one respondent said: ‘*discussing early with the family about palliation*’ (EP 10). Participants also thought that with attention to frailty, the delivery of care might more likely occur in the appropriate setting. They referred to potentially optimizing care and assisting frail patients to continue living at home in the community. Furthermore, some participants reflected on the need for nursing home staff and paramedics’ attention to frailty status when deciding about transferring residents to the ED. As one participant expressed: “*nursing home staff … have to be made more aware of these things, I’m not sure how much right they [paramedics] have to say: ‘ok, we assess this patient as frail and have got nothing or medical going on, and the patient does not need to come to the hospital*’” (EP 12).

Participants noted that the awareness of frailty status in patient care, and addressing contributory and risk factors for adverse outcomes, could also result in improvements in health service use, such as reducing ED presentations and hospital admissions. As one EP said: ‘*less falls... would mean they would have less broken bones or other reasons to come to ED …. or hospitalization*’ (EP 14). Appropriate decisions with avoidance of unnecessary ones also represented addressing futility, that is measures which are not indicated. As one interviewee expressed: '*only admitting patients who truly need to be admitted and ensuring as best we can that patients who can be managed in the community are so, with the appropriate supports in place*’ (EP 16).

Sub-theme 3.2: EPs Advocated for Resources and Training Related to Frailty.

Participants referred to the frailty-related needs for improved resource provision and application to progress with collaborative multidisciplinary care. As one respondent said: ‘*having the empathy and the resources to be able to support them … especially upon discharge and getting home....It’s something that we do lack to some degree, in that …we are focused more on acute medicine*’ (EP 7). References to government and organizational support for the potential progress were made, such as with community resources including equipment, follow-up clinics, and access to relevant HCWs. As one EP stated: ‘*services that may be provided by the State government in terms of tools, clinics, dietitians etc*’ (EP 6). One participant pointed to improved funding for frailty-related assessment of nursing home residents; this could also represent improved cost efficiency by service provision at the appropriate site: ‘*if there was … an appropriate sort of funding stream to have patients assessed in the nursing home by an appropriately skilled clinician…*’ (EP 16).

Further to participants alluding to the growing number of older patients attending the ED as well as the perceived lack of knowledge and skills on frailty, one respondent referred to the case for related training for EPs and collaborating HCWs to progress with the ED care of frail patients: ‘*Training, I would say for emergency physicians and geriatricians …*’ (EP 15).

## Discussion

Our qualitative study is relatively novel, exploring solely EPs’ perspectives on frailty, in contrast to other studies where responses from EPs combined with those of other HCWs were analyzed [14-16, 30-33]. We found most EPs perceived frailty as a chronic condition, not a priority of emergency care, and seldom referred to the term. They reported using clinical skills to detect components of frailty rather than a formal method with validated tools to identify a unified conceptual syndrome. Most participants referred to lacking knowledge of frailty, but the need for training on frailty was recognized by a minority. Going forward, EPs envisioned progress with formally detecting and addressing frailty, especially outside the ED setting, for patients to benefit in their quality of life and wellbeing, and through avoidance of futile care. EPs referred to their responsibilities including communication and collaboration.

Overall, our participants’ understanding of frailty in older people resembled that of HCWs in other studies, referring to associated factors and their inter-relations as well as vulnerability [5, 15, 16, 34-37]. Close to half of our participants demonstrated empathy in their understanding of frail patients’ struggles, such as patients’ feelings of isolation and depression. These perceptions related to consumers’ perspectives [38-40]. EPs self-acknowledged lacking clinical knowledge on frailty, like other HCW groups namely GPs, orthopedic surgeons, speech therapists, and mixed [16, 17, 30, 34, 36]. This is likely related to our EPs’ reported lack of clinical training on frailty and usage of the term. Participants identified barriers to formally addressing frailty, including clinical orientation, time, and work pressures as well as lack of knowledge, training, and resources, which have been reported previously [16, 30, 37]. Our participants’ references to the application of gestalt for detecting frailty as opposed to formal assessment were also identified in other qualitative studies with other HCWs from Australia and other countries [5, 15, 17, 30, 34]. The strategy for frailty recognition through skills developed from clinical experience and knowledge from different sources, instead of focused training, relates to the ‘mindlines’ system described by Gabbay and le May [41]. Without the use of a formal method to assess for frailty, however, the diagnosis may be missed or delayed in those with earlier disease and the severity overcalled in others. This could contribute to decisions or treatments that are not in the patient’s best interest.

References similar to our ED consultants’ have been made by HCW groups to the relatively recent adoption and application of the concept of frailty in clinical practice [16], as well as limited education and training on frailty [16, 30, 42]. The need to improve holistic or comprehensive care of frail older adults has been referred to by health professionals in other studies [15, 43], and reflected in consumer perspectives [44, 45]. To address this, undergraduate, specialization, and professional development programs need to continue progressing with the incorporation of frailty education [30]. For example, the recent inclusion of attention to frailty in the emergency care of older patients by the Australasian College for Emergency Medicine occurred in 2020 in its policy for the care of older people in the ED [8], and in its Fellowship training curriculum in 2021 [46, 47].

Our participants did not mention working within a frailty-related ED model of care for frail older persons such as those in place in Singapore with the frailty-ready EDs system [11], and in the United States of America with the Geriatric Emergency Department collaborative model [48]. Such systems of care have been evolving worldwide in the past decade [11, 49-51]. Our EP respondents concur with HCWs in other qualitative studies on the potential benefits of frailty identification and collaborative multidisciplinary patient-centered care [15, 30, 43], including understanding the frail patient’s needs and risk [16], improved quality of life in different treatment circumstances (e.g., palliative care [52]), and measurable outcomes such as hospital admission avoidance [32]. Evidence has been growing about the impact of frailty awareness in the ED, to a greater extent presently on associations of frailty status as a predictor for patient outcomes (such as admission and mortality) [2, 53, 54] compared to patient and system benefits of frailty-related interventions in the ED [10, 51, 54-57]. For example, two studies set in Singapore within the Emergency Department Interventions For Frailty (EDIFY) program described favorable results with a multicomponent frailty intervention by geriatric HCWs for older patients prior to ED discharge [55, 56], with decreased admission rates in the first study [55], and reduced ED re-presentations as well as better preservation of function in the second [56].

Limitations

Data were collected within one health network without a formal frailty-related ED model of care in place. Data from a broader catchment with diversity in frailty-related ED care models would likely generate different results. EPs’ perspectives on frailty in different health jurisdictions would vary as evidence-based knowledge on frailty, and related implementation of frailty screening and management evolve at variable rates. While more of a note on context rather than a limitation, the interviews were conducted in 2018-2019 prior to the COVID-19 pandemic; workplace knowledge and culture have shifted post-pandemic in complex and unpredictable ways. While the interviewer (JS) took steps to be reflexive, this may not have altogether mitigated the influence of his professional background and relationship with participants on data collection and analysis.

## Conclusions

Our qualitative exploration of perspectives on frailty among EPs has identified frailty as not being a priority of emergency care, with perceived barriers including a focus on acute care, time and workload pressures, and lack of knowledge and resources. Nevertheless, participants were open to the benefits of frailty interventions for frail patients. Not only is there a need for frailty education to become commonplace within undergraduate and specialty training programs, but there is also a need to build the evidence base to convince clinicians of the benefit of frailty awareness and move toward the implementation of promising new practices such as frailty screening. Leadership in the ongoing development of frailty-related systems of ED care is indicated as well. While evidence is emerging, there remain important areas for future research, such as investigations across diverse jurisdictions with various models of ED practice. However, this research provided an important snapshot into the perspectives of EPs on frailty within one health network. As health systems grapple with an aging population, our findings could contribute to facilitating the implementation of frailty screening, assessment, and corresponding frailty-related interventions in the ED.

## Appendices

**Table 2:** Emergency Department Physician Interview Guide. Frailty and Frailty Screening

Questions in the interview guide
What does frailty mean to you?
What are your general thoughts on the term itself? (Connotations?)
Do you ever refer to the term frailty in your practice? (Colleagues? Patients? Families?)
What do you think are the experiences of frail persons?
Thinking of what frailty means to you, can you offer a definition of frailty from a clinical perspective?
When would you say that someone is frail?
What is your understanding of how frailty develops?
How does it progress?
What, if anything, can be done to prevent people from becoming frail?
What can be done once a person is frail?
What can emergency department physicians (and other health care providers) do to improve care of pre-frail and frail persons?
Can you describe your attitude toward the concept of frailty screening? Under what circumstances if any do you feel it would be useful? Under which time points and settings would it be most useful?
What would make a screening tool most feasible for use within your practice context?
If a tool indicated a patient to be frail, what would the logical next steps be for you? Would this change how you already practice? (If so, in what ways?)
In relation to the transfer of nursing home residents to the emergency department, what role for decision-making do you see with frailty screening in the emergency department?
Moreover, with regards to the residents’ transfer from the nursing home to the emergency department, what role for pre-hospital decision-making can you suggest with frailty screening at the nursing home?
